# Activate Your Health, a 3-year, multi-site, workplace healthy lifestyle promotion program: study design

**DOI:** 10.1186/s12889-019-7393-x

**Published:** 2019-08-19

**Authors:** Thiffya Arabi Kugathasan, François Lecot, Suzanne Laberge, Jonathan Tremblay, Marie-Eve Mathieu

**Affiliations:** 1 School of Kinesiology and Exercise Sciences, Faculty of Medicine, Université de Montréal, P.O. Box 6128, Downtown Station, 2100 Édouard-Montpetit, Montreal, QC H3C 3J7 Canada; 2 Sainte-Justine University Health Center, Montreal, QC Canada

**Keywords:** Workplace, Health promotion program, Lifestyle habits, Lifestyle change

## Abstract

**Background:**

Workplace Health Promotion Programs (WHPP) have been shown to be an efficient way of improving workers’ health. These programs can be incorporated in the worker’s daily schedule and improve their productivity at work. Improving employees’ health also benefits the employers by increasing their return on investment and lowering healthcare costs. The *Activate Your Health* program, created by Capsana in 2015, is a WHPP targeting multiple lifestyle habits for a three-year period. This WHPP includes tailored web-based interventions and the support of different health professionals throughout the years. We hypothesize that this approach will yield long-term lifestyle changes. The objective of the current paper is to describe the *Activate Your Health* program’s design.

**Methods/design:**

Eleven companies are taking part in this WHPP and had to choose among five different options of this program and all their employees were encouraged to participate. Each option differs by the number and type of interventions included. The limited option, which is considered the control group, only consists in completing a questionnaire regarding their health status, lifestyle habits and behaviors. On the other end, the extensive option receives a combination of multiple interventions: online menus, health challenges, support in creating a healthy work environment, coaching by health professionals (nurse, nutritionist, and kinesiologist), health screening and flexibility assessment, online resources, social health platform, and activity tracking. The remaining options are in between these options and vary by the amount of intervention. Baseline data are already gathered; two other data collection periods will take place after one and 2 years into the program. The primary outcomes of the current program are physical activity and fitness measures, nutritional data, smoking habits, stress and intention to change.

**Discussion:**

The *Activate Your Health* program will allow us to compare which combinations of interventions are the most effective. It is expected that the extensive option will be the most advantageous to improve lifestyle habits. The results will indicate the strength and weakness of each intervention and how it could be improved.

**Trial registration:**

Clinicaltrails.gov, registration number: NCT02933385 (updated on the 26th of March 2019, initially registered on the 5th of October 2016).

## Background

The World Health Organization states that chronic conditions are the leading cause of death and disability worldwide [1]. More than one out of every five Canadian lives with one of the following chronic conditions: cardiovascular disease, cancer, chronic respiratory disease, or diabetes [2]. These four chronic diseases represent 67% of annual deaths [3].

In Canada, 50% of workers live with a minimum of one chronic condition [4]. This situation increases absence rates due to functional limitations and creates a financial burden to the employers [5]. Moreover, poor health and unhealthy lifestyle habits were observed to decrease productivity [6]. Thus, decreasing chronic conditions is crucial. This could be achieved by adopting healthy lifestyle habits [7, 8]: i.e., being physically active, eating healthy, limiting alcohol and tobacco, and having good sleep habits, [9] which can prevent at least 70% of major chronic diseases [10]. Engaging in 180 min of moderate intensity physical activity (PA) per week, which is roughly the recommended amount of PA, decreases the risk of mortality [11]. However, it has been noted that the Canadians lack compliance to the recommendations for a healthy lifestyle. For example, 78% of Canadians are not sufficiently active and 60% have poor eating habits [12]. It is also encouraging to note that partly fulfilling the recommendations could have beneficial effects on health. For example, a sedentary individual who does any PA would benefit from a decrease in mortality risk [11].

Some of the reasons for failing to comply with these healthy lifestyle recommendations are a lack of time, social norms, built environment and cost [13]. As workers spend roughly 8 h per day at work [14], a program targeting changes in the lifestyle habits could begin at the workplace. To do so, some companies have started seeking the help of health specialists to improve their workers’ lifestyle habits and working environment by implementing a workplace health promotion program (WHPP). Studies indicate that these programs are beneficial for the workers’ physical [15, 16] and mental [17, 18] health, but also improve their work performance by increasing productivity [19, 20] and decreasing absenteeism [21]. These changes indirectly reduce health-care costs [22] and increase “return on investment (ROI)” [19, 22]. Hence, WHPP would advantage the workers, employers and the society as a whole [23–25]. However, this type of program needs to be designed properly. For example, a WHPP offering only a health-related questionnaire and/or an online platform does not lead to a successful outcome [26]. Moreover, to detect the population health effects and to yield a positive ROI, a minimum of 3 years of implementation is suggested [26].

Capsana is a Canadian organization dedicated to promoting healthy lifestyle habits, and preventing and managing chronic diseases in the workplace for the past 25 years. In 2015, Capsana [27] created a program called “*Activate Your Health*”. This WHPP allows workers to adopt healthy lifestyle habits in their workplace with web-based and tailored interventions along with personalized advice over a 3-year period. The program also helps workers identify their risk factors associated with chronic diseases. In addition, it also assists the employers in changing the culture of health within the company in the goal of supporting the employees in achieving a better health profile and lifestyle habits. The eventual goals of the evaluation component of the *Activate Your Health* program are to evaluate the effectiveness of this WHPP on the short and long term, investigate which workers would benefit the most, compare the responses to the different interventions, and identify the challenges associated with the implementation of this type of program in the participating workplaces. We hypothesize that the option targeting several lifestyle habits at once will yield the biggest improvement in health and work-related parameters. The aim of the current paper is to describe the *Activate Your Health* WHPP.

## Methods/design

### Study design

The *Activate Your Health* program is an already-existing three-year program that was designed and implemented by Capsana in Canada (Quebec). This quasi-experimental study with an exploratory framework takes into account previously identified limitations regarding WHPP and interventions that previously have been shown to be beneficial [26, 28, 29]. The baseline data was collected between December 2016 and July 2018. The employees will be re-evaluated one and 2 years into the program. There was no specific risk associated with participating in this program other than the ones associated with the adoption of an active lifestyle. The Health Research’s Ethic Committee of Université de Montréal approved this study (16–063-CERES-D (1)). The study was registered on Clinicaltrials.gov on 16th of October 2016, and was updated on the 26th of March 2019 (NCT02933385).

All the gathered information was kept confidential and was not shared with any employers. The employers received only an overall report regarding the health of their employees without any given individual’s portrait. Participating employees were aware of this and also knew that the data collected throughout the *Activate Your Health* WHPP would be part of a research study. Only certain Capsana’s employees associated with data management and the research team had access to this information.

### Recruitment of participants/study population

Through phone calls and in-person meetings, Capsana contacted the different companies to participate in their *Activate Your Health* program. Eleven companies are taking part in the current project. Among the participating companies, six were related to banks, two were related to financial markets, one was a graveyard parish, one was related to workplace health safety and security, and one was a marketing firm. The participating companies encouraged their employees to take part in this WHPP and participation was on a voluntary basis. Only pregnant women could not partake in the program. After an information session about the *Activate Your Health* WHPP and motivating their willingness to participate, an email was sent out to all the employees. Those who completed the online consent form (written) were able to fill the self-administered questionnaire.

### Intended sample size explained

Based on recruitment experience for similar workplace interventions, financial support available and pilot data yielding significant benefits for the employees and their organizations with 656 participants, this program had the potential to enrol 6000 employees [30]. Therefore, *Activate Your Health* included as many employees as possible. Also, the purpose of this program is to evaluate for the first time the overall impact of the different options on a lot of health-related variables and lifestyle habits therefore the goal was to involve as many participants. The data collected for this program will thus be used for the first time to evaluate this program and will serve as a model for sample size calculation of future studies.

### Study intervention/allocation to interventions

Capsana and their sponsors are offering the interventions throughout the year and at the same time of year throughout the 3-year period (ex: a company that received an intervention in January 2017 will receive the same intervention in January 2018, 2019). Table 1 summarizes the different interventions included in each of the five options (A to E) and the number of companies enrolled in each of them. Briefly, option A had the most extended number of interventions, and involved numerous web-based and innovative tools to improve lifestyle habits. Moreover, some interventions in option A and B were tailored and personalized to the participants’ needs while participants in option E will not receive any interventions other than the informational/motivational sessions at baseline and at the end of each year (control group). There was no concomitant care or interventions that were prohibited while taking part in this program.

**Table 1:** Details on the interventions included in each option (*n* = 11 companies)

Interventions	Options				
	Extensive				Limited
	A (*n* = 1)	B (*n* = 1)	C (*n* = 8)	D (*n* = 0)	E (Control) (*n* = 2)
Personalised online menus	X	X	X	X	
Support in creating a favourable environment	X	X	X	X	
*Health Challenge*	X	X	X	X	
Conferences	X	X	X		
Coaching	X	X	X		
Closing events	X	X	X		
Health screening and flexibility assessment	X	X			
CANRISK questionnaire	X	X			
Distribution of publications	X	X			
Social health platform	X	X			
Activity tracker	X				
*Optional: Quit to Win! Challenge*	X	X			

The companies had to choose one out of the five options offered by Capsana. Most of the companies (*n* = 8) chose option C and none picked option D. One company chose two different options (one group was placed in option A while the others in option E). One company won a promotional draw and Capsana placed it in one of the five options (Option E). In-person interventions took place at the workplace whereas the web-based ones were accessible anywhere.

Each company started the interventions at a different time point as each company differed by the administrative work necessary for the implementation of such program. Employees were unaware that other companies were participating in the *Activate Your Health* program and that the others could have a more or less beneficial option than theirs. Participants could also withdraw from the program at any given time. Participants are welcome to communicate with the research team if they have any questions/concerns during the program. Participants are also questioned at the end of the study regarding any adverse effects that took place during the study period. An employee from Capsana visited at least once a year each company to improve the adherence that will also be monitored by completing a questionnaire at the end of each year. The research team assisted annually to at least one data collection session and one of the interventions offered in this WHPP.

### Data collection tools

#### Self-administered questionnaire – year 1, 2, and 3

All employees taking part in *Activate Your Health* are asked to fill out an online self-administered questionnaire that is based on an adaptation of existing questionnaires, lifestyle guidelines and opinion of health experts. Participants are asked to provide basic socio-demographic, anthropometric, health, medication and work information and extreme values are checked before inclusion. Lifestyle habits are also auto-reported: PA (frequency, duration and intensity), eating habits, smoking, alcohol consumption, sleeping habits, and stress level. As the *Activate Your Health* program aims to modify the employees’ lifestyle habits, their intention to improve their habits is also evaluated. Once the questionnaire is submitted to Capsana, each of them is assigned a questionnaire ID to anonymize the data. The data are kept for 7 years by the Data Monitoring Committee. The Data Monitoring Committee is led by the principal investigator (MEM) in collaboration with one member of Capsana. It supervises data collection and analyses. For any further details please contact the principal investigator. After which, a total health score is calculated on 100 points: the higher the score, the better their health (100 equals optimal health and lifestyle habits). This score and action plan are sent to the employees personally.

### Detailed description of each intervention

#### Personalized online menus – year 1, 2, and 3

Each employee has access to an online platform called *SOScuisine*: it offers weekly menus, discounts, recipes and grocery shopping lists adapted to the employee’s preferences and health goals (improve cholesterol, manage diabetes, weight control, etc.).

#### Support in creating a favorable environment – year 1, 2, and 3

Health professionals visit each participating companies to host and present an information booth on how to create a favorable environment to improve their employees’ health (PA, nutrition and life balance). For example, they show the employees how to be active around their workplace, and highlight the healthiest menus in their cafeteria or vending machine in order to improve their eating habits.

#### Health challenge – year 1, 2, and 3

Employees are invited to participate individually or as a team in a 4-week activity called *Health Challenge*. It is publicized at their workplace and participation is voluntary. This intervention aims to guide companies and their workers towards better eating habits (i.e., five servings of fruits and vegetables/day), PA level (be physically active 30 min/day) and mental wellness (take time to relax). Capsana guides and motivates the employees who take part in this challenge. Employees who successfully fulfill the challenge receive an incentive (i.e., a sport bag). The participating company managed this intervention.

#### Conferences –year 1, 2, and 3

Different health professionals (nurse, kinesiologist, physician, psychologist, etc.) present a 60-min conference each at the work site of participating company at different time points into the study. The purpose of these sessions is to inform and educate employees on the importance of adopting and maintaining a healthy lifestyle. The health professional also gives tips and ideas to help employees achieve these goals. More conferences emphasizing mental well-being are planned.

#### Coaching – year 1, 2, and 3

Employees are offered assistance in maintaining or improving their lifestyle habits. They have the opportunity to talk, through the phone or in person, to a nurse and receive personal motivational coaching to enhance, among others, psychological health, well-being and lifestyle habits, and to reduce stress (Year 1). The following years, they will have the opportunity to talk to a nutritionist (Year 2), and to an exercise specialist/kinesiologist (Year 3).

#### Closing events – year 1, 2, and 3

At the end of each year, at the work site, employees will take part in a closing event organized by Capsana. An example of this is “Jeux Spin [31]” which consisted of various recreational activities such as soccer in an inflatable structure. It is to note that for logistic reasons, these events are offered to the participating companies (not just the participants of the *Activate Your Health* program). These events aim to congratulate the employees for taking control over their own health and motivate them to maintain their acquired/improved habits.

#### Health screening and flexibility assessment – year 1, 2, and 3

A team of nurses meets the employees individually to screen for their risk factors. The goal of this intervention is to identify any abnormal blood profile (unfasten state). It serves as an indicator and does not replace a regular blood test. If any abnormal results were found, employees were encouraged to consult their family doctor in order to do a follow-up. The plasma HDL level and HDL/total cholesterol ratio (CardioChek P.A.®-LIT001539, QC, Canada) and glucose concentration (Contour®Next - 85,303,759, New Jersey, US) are measured. Blood pressure is manually taken using the conventional auscultatory method (stethoscope: 3 M™ Littmann® Classic II S.E-MMM2201, US; sphygmomanometer: Welch Allyn & Tycos model, Boston, US). Employees are asked to stand erect in order to take their height using a vertical stadiometer (Seca213, Hamburg, Germany), and weighed using a scale (Tanita-BF-350, IL, US). Waist circumference is measured at the iliac crests using a measuring tape. The “sit-and-reach” test (Sit and Reach Test Tester- EN-121085, NY, US) is performed to determine posterior chain flexibility. The results of these tests are available to the employees at the end of their session, and the nurse gave appropriate health advice to improve their health profile.

#### CANRISK questionnaire – year 1, 2, and 3

During the screening session, participants also complete the CANRISK [32] questionnaire to determine their risk of developing prediabetes or diabetes. It consists of 12 multiple-choice questions, resulting in a score that is associated with three risk categories: low (< 21 points), moderate (21–32 points), and high (> 32 points). Employees also receive this score with the interpretation.

#### Distribution of publications – year 1, 2, and 3

During the conferences, screening and informational sessions, interested employees receive Capsana’s documentation (flyers/brochures/booklets/etc.) on chronic diseases (diabetes and cardiovascular health), mental health, medication, etc. These resources are also available on Capsana’s website.

#### Social health platform – year 1, 2, and 3

Each employee has access to a Social health platform (Sprout [33]), which allows employees to interact with one another. They can set goals, create an interest group, and challenge their colleagues and themselves. For example, an employee could challenge a colleague to take the stairs as often as they can.

#### Activity tracking – year 1

Capsana offered an accelerometer bracelet (Vívosport, Garmin, 2017) to 250 employees selected at random and invited them to track their own physical activity information through the Garmin website or phone application.

#### Optional: Quit to Win! Challenge

This intervention is part of the provincial program (« J’arrête, j’y gagne! ») initiated by Capsana [34]. Only the interested companies take part in this challenge. It is promoted at the workplace and information booth with documentation available to all the employees. Those who are interested are invited to stop their tobacco use for 6 weeks, and Capsana guides and assists the employees throughout. Employees are guided through this challenge and the winner receives an incentive such as a trip to Jamaica.

### Outcomes measures

Yearly assessments of a broad range of physical and psychological health outcomes will be performed to assess yearly changes and changes from baseline of each option.

#### Primary outcomes

Physical activity parameters, eating habits, smoking habits, alcohol consumption, sleeping habits, stress level and intention to improve these habits are measured.

#### Secondary outcomes

Waist circumference, weight, height, body mass index (BMI), plasma HDL and HDL/total cholesterol ratio, blood glucose concentration, systolic and diastolic blood pressure, flexibility level and risk factor profile are assessed.

### Statistical analyses

In future analyses, option D will be excluded as no company selected this option. To examine if the different options have similar baseline characteristics, i.e., to verify the homogeneity between the four options for the selected baseline variables one-way ANOVA analysis with the effect size (partial eta squared) and Chi-square test with Cramer’s V effect size will be used. Future studies will also explore the effect of each option on all outcomes. For continuous variables, mixed models for repeated measures with two factors will be used to analyze the effect of time and option on each outcome following one and 2 years of measurements. As for the categorical variables, generalized estimating equations (GEE) will be used. Possible confounders will be considered. Future studies will also take into account the cluster effect as the participants within the same company undergo the same implementation process and working environment, and therefore individual results from a same participating company could be dependent on each other. In future studies, intent-to-treat analysis will be used as well as multiple imputations. In addition, a particular consideration will be given to the sex and weight category of the participants.

## Discussion

There is a growing body of literature confirming that WHPPs are advantageous for employees’ physical and mental health, and this has been confirmed objectively and/or subjectively [35–38]. WHPP also improves employee’s lifestyle habits, which are related to the presence of chronic diseases [9, 10]. These programs benefit the individual, company and the society [23–25]. The current study describes a recently implemented program called *Activate Your Health* that aims to improve employees’ lifestyle habits through a WHPP in the working environment. The data collected through this WHPP will be used for the first time to evaluate the efficacy of this large-scale program.

One of the potential strengths of the *Activate Your Health* is that it targets multiple modifiable lifestyle habits and biometric measures associated with a decrease in the risk of cardiovascular disease and stroke [39]: eating healthy, being physically active, discontinuing smoking, and maintaining a healthy weight, blood pressure range, blood cholesterol and glycaemia. Recent studies in the field targeting at least three habits [40, 41] were observed to be effective. Therefore, it is expected that multiple lifestyle habits in one option would lead to greater health benefits for employees. Adherence rates in WHPP are generally in the low range [42] (< 50%), and web-based interventions, when tailored, were observed to be effective [43] only for a short period of time after the study period [44]. In the current study protocol, the web-based tailored interventions are accompanied with the support of different health professionals (nurse, nutritionist and exercise specialist/kinesiologist) throughout the 3-year period. Therefore, the adherence rate and effectiveness of the options A, B and C are expected to be higher. In addition, a study [45] with a similar approach, i.e., targeting six health behaviors, but differing in the study population (male participants at high risk of cardiovascular disease without a control group), the variety of interventions offered (3.5 h of health promotion class), and study duration (6 months) led to an increase in physical activity, improved stress management, diet control, blood pressure, total cholesterol and BMI, which are some of the variables that will be included in future studies. Therefore, *Activate Your Health* might lead to similar or better results as it regroups any health profile of employees, has a longer study period, provides different interventions that are web-based, and support the company in creating a favorable work environment.

One of the advantages of the *Activate Your Health* program is the varying number of interventions, which allows us to compare the effect of different combinations of interventions on health and work-related variables. We did exclude the option D as none of the companies chose that option. We can deduct that such an option might be less appealing for companies. It would have been interesting to compare the option A, which contains all the interventions targeting eating and PA habits vs. the option D, which had only interventions improving eating habits. Nevertheless, we do have the limited option (control group) that did not receive any interventions to compare the effectiveness with the other options. Moreover, Nöhammer et al. (2010) suggested that WHPP could benefit from documenting the employee’s perspective of the interventions. At the end of the current WHPP, a questionnaire regarding each intervention will be sent to all the employees, in order to study their perspective. Also, throughout the year, Capsana also supports each company in the creation of a favorable environment by providing advice and/or help [46]. Modifying the work environment by decreasing unhealthy food available at the work site within the company, for example, could have positive impact on the employees’ health and the organization as a whole [46].

Some limitations should be considered. The data collection method consists of filling questionnaires. Most of the variables of interest, such as PA level and eating habits, in the current program and its variations come from these self-reported answers. Objective measures such as accelerometer data could have been used to identify the changes that are taking place within the different options. Moreover, participants who have successfully changed their lifestyle habits will be more inclined to remain actively involved in the *Activate Your Health* program, and to be reassessed, which was the case in another study [47]. In addition, Nilsen et al. [47] (2014) noted the majority of their participants with diabetes who dropped out were the participants with a poor life satisfaction and needed help with their lifestyle habits. Taking these observations into account, specific analysis will be performed to examine this aspect in *Activate Your Health*.

In the *Activate Your Health* program, among other advantages, assessing employees’ health using health-related assessments, targeting several lifestyle habits at once, offering tailored interventions, providing social platforms, improving the workplace environment, and offering a three-year program would most likely improve employees’ lifestyle habits [46]. Future studies will also focus on gender and body weight status response. In the end, the goal would be to articulate which type of intervention works best and for whom, to consolidate but also to improve interventions.

## Data Availability

Not applicable. The results will be communicated to the participants and to other relevant groups via publications (open access will be prioritized) and presentations, including webinars.

## Abbreviations

BMI: Body mass index
PA: Physical activity
ROI: Return on investment
WHPP: Workplace Health Promotion Program
